# An Unusual Case of an Acquired Esophageal-pulmonary Fistula Caused by Hodgkin Lymphoma

**DOI:** 10.7759/cureus.2589

**Published:** 2018-05-07

**Authors:** Juan J Gonzalez, Lakshmi Talasila, Lisa Ochoa, John Youssef, Orimisan S Adekolujo

**Affiliations:** 1 Internal Medicine Department, Mclaren Flint, Michigan State University, Flint, USA

**Keywords:** hodgkin's lymphoma, esophageal-pulmonary fistula, esophageal stent, chemotherapy

## Abstract

Bronchoesophageal and tracheoesophageal fistulas are an uncommon but well-documented complication of Hodgkin lymphoma (HL). To our knowledge, a fistula directly connecting the esophagus with the lung (esophageal-pulmonary fistula) and resulting from HL has not been reported in the literature. We present a case of HL complicated with an esophageal-pulmonary fistula. The early recognition and treatment of an esophageal-pulmonary fistula in patients with HL are imperative since, with appropriate treatment, HL complicated with an aero-esophageal fistula has the same prognosis as those without one, unlike the dismal prognosis in esophageal and lung cancer. Endoscopic esophageal stenting followed by chemotherapy is the preferred treatment approach. This leads to the healing of the fistula and the prolongation of patient survival.

## Introduction

Acquired aero-esophageal fistulas are a rare, but well-documented complication, of Hodgkin lymphoma (HL). Most are either bronchoesophageal or tracheoesophageal fistulas [1-3]. Upon an extensive literature search, we were unable to find any report of a fistula directly connecting the esophagus with the lung (esophageal-pulmonary fistula). We present a rare case of an acquired esophageal-pulmonary fistula secondary to HL.

## Case presentation

**Figure 1:**
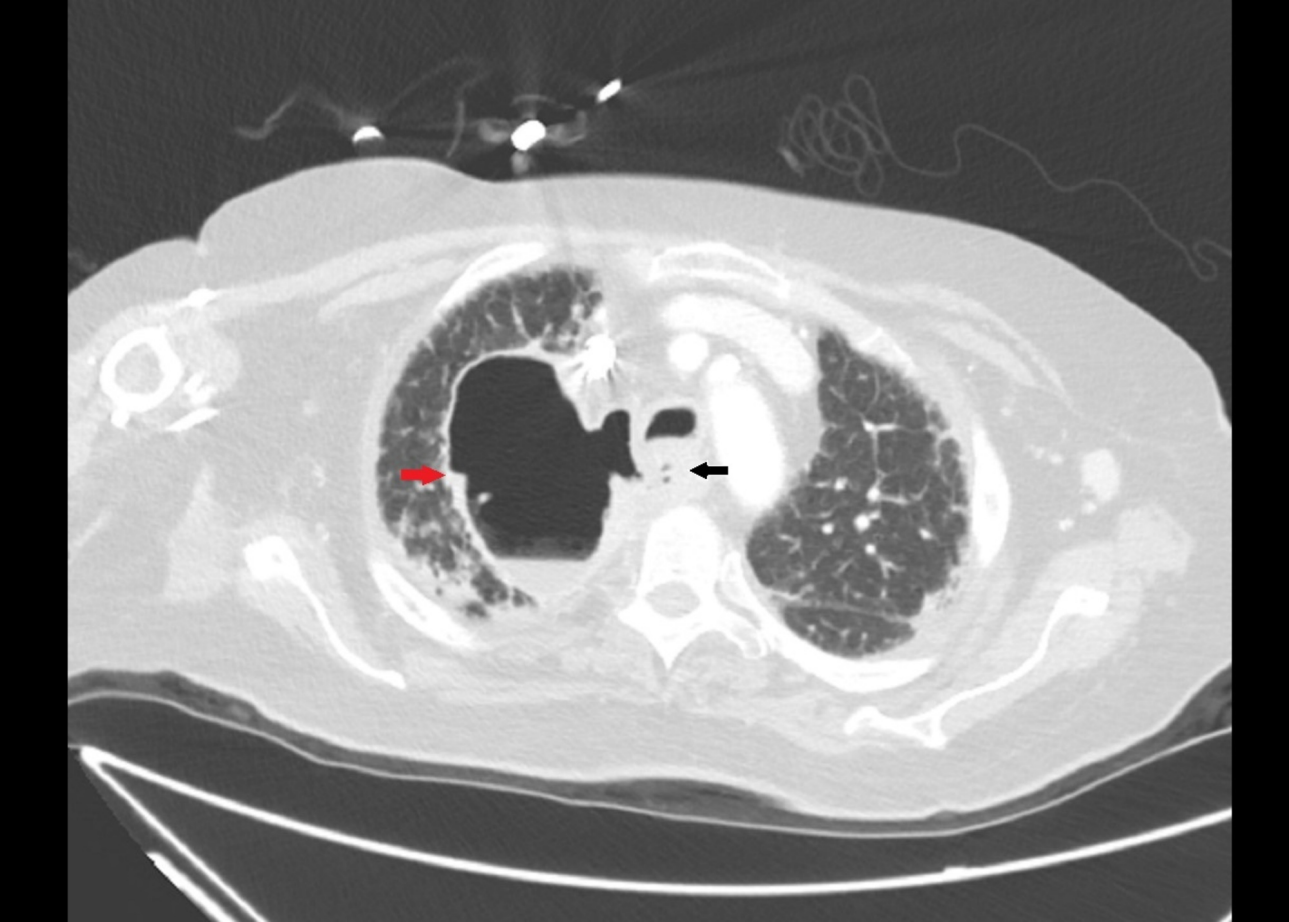
Esophageal-pulmonary fistula Computed tomography of the chest with contrast appears to show a fistulous communication with the esophagus (black arrow) along with a right upper- lobe cavitary lesion (red arrow) with irregular wall thickening.

**Figure 2:**
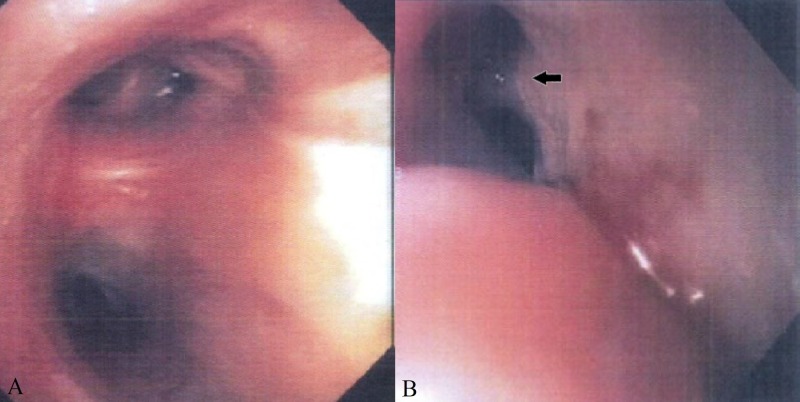
Bronchoscopic and esophageal anatomy view A) Bronchoscopy: Bilateral bronchial tree and all subsegments showing no evidence of fistula; B) Upper Endoscopy: Features suggestive of a fistulous tract (black arrow).

**Figure 3:**
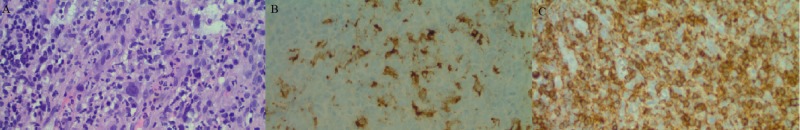
Histopathology of esophageal tissue A) Esophageal biopsy from the fistulous tract showing highly atypical cells; B) Immunostaining positive for CD30; C) Immunostaining negative for CD45.

A 72-year-old Caucasian female with a history of stage II lymphocyte-rich classical Hodgkin lymphoma was treated with combination chemotherapy, including doxorubicin, bleomycin, vinblastine, and dacarbazine (ABVD) with resultant remission. After seven years in remission, she developed persistent leukocytosis and anemia. Positron emission tomography-computed tomography (PET-CT) done for the evaluation of recurrence showed a new, enlarged left axilla, right paraesophageal, right precarinal, aortopulmonary, portohepatic, peripancreatic, and pericaval lymph nodes. The report of the biopsy of the enlarged right paratracheal lymph nodes via mediastinoscopy was consistent with HL. A diagnosis of recurrent stage III Hodgkin lymphoma was made. She was treated with gemcitabine, vinorelbine, and doxorubicin. Although imaging showed a response to therapy, chemotherapy was discontinued after four months of treatment due to significant functional decline and poor performance status. One year after the discontinuation of chemotherapy, she presented with a productive cough and persistent choking, preventing oral intake and marked weight loss. A physical examination revealed pallor, signs of dehydration, and bibasilar crackles. Due to a high suspicion of aspiration pneumonia, she was admitted and started on intravenous antibiotics. Computed tomography (CT) of the chest (Figure 1) and CT soft tissue of the neck (Figure 1) with intravenous (IV) contrast showed a 7.4 x 5.6 cm cavitary lesion involving the right upper lobe with irregular wall thickening and a fistulous communication with the esophagus. Bronchoscopy with an evaluation of the bilateral bronchial tree and all subsegments showed no evidence of a fistula (Figure 2A). Bronchoalveolar lavage and cytology were negative. Upper gastrointestinal endoscopy revealed a 4-cm long stricture in the proximal esophagus and features suggestive of a fistulous tract (Figure 2B). The histopathology of multiple biopsies obtained from the fistulous tract showed highly atypical cells with immunostaining positive for CD30 and PAX5 (Figure 3), consistent with the involvement of esophageal tissue with Hodgkin lymphoma. Esophageal stent placement was planned but could not be performed due to severe thrombocytopenia. Percutaneous endoscopic gastrostomy was considered but the patient declined and eventually opted for hospice care. She died a month after the initial presentation.

## Discussion

The majority of acquired aero-esophageal fistulas are associated with neoplastic diseases. The most common cause is primary esophageal or lung cancer [1,3]. Other causes include prolonged intubation, corrosive ingestion, granulomatous mediastinal infections, prior surgery, or trauma [4]. Acquired aero-esophageal fistulas due to lymphoma have been uncommonly reported, and the localization is usually between the trachea or bronchus and the esophagus [2]. This case is atypical, as the esophageal fistula communicates directly with a cavity in the right lung, a feature that has not been described before for HL. In this case, communication of the fistula with the lung delayed potential therapeutic interventions due to the need for multiple invasive procedures leading to a subsequent diagnostic delay. The most prevalent cause of fistula formation due to HL is radiation therapy with or without chemotherapy [2]. Another cause, as demonstrated in the index case, is a direct extension of the lymphoma into the esophageal tissue [5]. Inflammatory cytokine production by the lymphomatous cells contributes to local tissue necrosis and the invasion of adjacent structures [6]. Unlike in esophageal or lung cancer where fistula development represents a grave prognosis, patients with HL complicated with a fistula have similar survival to patients without this complication after healing of the fistula [2,7]. Without treatment, the mean survival time is only one to six weeks. Treatment focuses on the healing of the fistula and the prolongation of patient survival [3]. Currently, endoscopic esophageal stenting is the preferred approach followed by an attempt to provide effective chemotherapy, which is challenging in the presence of pulmonary sepsis and malnutrition [8]. Other options include gastrostomy tube placement and surgical treatment as the last resort [9]. Esophageal stenting is less invasive and can result in fistula healing, resolution of pulmonary sepsis, and improved nutritional status [8-9]. In the current case, esophageal stenting was not possible due to severe thrombocytopenia and malnutrition. The placement of a feeding gastrostomy may also result in sustained nutritional resuscitation and the control of pulmonary sepsis for fistula healing, but our patient refused this therapeutic approach.

## Conclusions

An acquired esophageal-pulmonary fistula is a rare complication of Hodgkin lymphoma. A high index of suspicion in patients with unexplained symptoms, such as a severe cough and choking, are important for the early diagnosis and institution of a potentially curative treatment.
